# Supplementary material for: MetaRibo-Seq measures translation in microbiomes
Source: Nat Commun. 2020 Jun 29;11:3268. doi: 10.1038/s41467-020-17081-z (PMC7324362; doi:10.1038/s41467-020-17081-z)
Supplement: Supplementary file 10 — Supplementary Data 7 [file 41467_2020_17081_MOESM10_ESM.zip › File2/Confidence_High_Taxonomy/210620_out.krona.html]

Javascript must be enabled to view this page.

members
magnitude
magnitudeUnassigned
count
unassigned
taxon
rank

210620\_out

3

superkingdom
3
2

3
phylum
1239

3
class
186801

186802
order
3

2
family
186803

genus
2
572511

2
species

SRS055966\_contig\_number\_contig-100\_6919.6920SRS147022\_contig\_number\_contig-100\_11291.73290
1737424

family
1
541000

1263
genus
1

165186

SRS1055034\_contig\_number\_contig-100\_25.26
1
species
